# A comparison of effects of scalp nerve block and local anesthetic infiltration on inflammatory response, hemodynamic response, and postoperative pain in patients undergoing craniotomy for cerebral aneurysms: a randomized controlled trial

**DOI:** 10.1186/s12871-019-0760-4

**Published:** 2019-06-01

**Authors:** Xi Yang, Jing Ma, Ke Li, Lei Chen, Rui Dong, Yayuan Lu, Zongze Zhang, Mian Peng

**Affiliations:** Department of Anesthesiology, Zhongnan Hospital of Wuhan University, 169 Donghu Road, Wuhan, Hubei China

**Keywords:** Scalp nerve block, Local anesthetic infiltration, Craniotomy, Postcraniotomy pain, Inflammatory response

## Abstract

**Background:**

The purpose of this study was to compare the effects of scalp nerve block (SNB) and local anesthetic infiltration (LA) with 0.75% ropivacaine on postoperative inflammatory response, intraoperative hemodynamic response, and postoperative pain control in patients undergoing craniotomy.

**Methods:**

Fifty-seven patients were admitted for elective craniotomy for surgical clipping of a cerebral aneurysm. They were randomly divided into three groups: Group S (SNB with 15 mL of 0.75% ropivacaine), group I (LA with 15 mL of 0.75% ropivacaine) and group C (that only received routine intravenous analgesia). Pro-inflammatory cytokine levels in plasma for 72 h postoperatively, hemodynamic response to skin incision, and postoperative pain intensity were measured.

**Results:**

The SNB with 0.75% ropivacaine not only decreased IL-6 levels in plasma 6 h after craniotomy but also decreased plasma CRP levels and increased plasma IL-10 levels 12 and 24 h after surgery compared to LA and routine analgesia. There were significant increases in mean arterial pressure 2 and 5 mins after the incision and during dura opening in Groups I and C compared with Group S. Group S had lower postoperative pain intensity, longer duration before the first dose of oxycodone, less consumption of oxycodone and lower incidence of PONV through 48 h postoperatively than Groups I and C.

**Conclusion:**

Preoperative SNB attenuated inflammatory response to craniotomy for cerebral aneurysms, blunted the hemodynamic response to scalp incision, and controlled postoperative pain better than LA or routine analgesia.

**Trial registration:**

Clinicaltrials.gov NCT03073889 (PI:Xi Yang; date of registration:08/03/2017).

## Background

Moderate to severe postoperative pain after craniotomy has an incidence as high as 80% [1]. Uncontrolled postoperative pain may contribute to increased intracranial pressure (ICP) and hypertension, which may be detrimental, especially for patients with cerebral aneurysms [2]. Therefore, postoperative pain control should be a priority for neurosurgical patients.

An increasing number of studies have suggested that multimodal pain treatment, which combines systemic analgesics and local anesthetics, optimizes pain relief and limits adverse effects of opioids [3, 4]. For example, scalp nerve block and local anesthetic infiltration of the scalp have been proposed to blunt hemodynamic response to craniotomy, decrease opioid consumption, and reduce postoperative pain perception [5–7]. Additionally, both scalp blocks and local anesthetic infiltration were recommended for enhanced recovery after surgery (ERAS) for oncological craniotomies, although the current evidence is not sufficient to create a standardized ERAS protocol for oncological craniotomy [8]. However, whether scalp nerve block or local anesthetic infiltration is more effective for analgesia has not been evaluated in craniotomy for cerebral aneurysms.

Surgery can initiate the inflammatory stress response. Surgical stress induces the migration of inflammatory cells that release cytokines, primarily interleukin (IL)-6, causing a local inflammatory response at the injured site. Then, when cytokines subsequently release into human plasma, systemic inflammation may occur that leads to an increase in C-reactive protein (CRP) and T- and B-cell activation in bone marrow and blood [9]. Later, compensatory anti-inflammatory cytokines, such as IL-10, are produced that cause a reduction in pro-inflammatory cytokine synthesis [10, 11]. Accordingly, acute-phase proteins, such as CRP, and cytokines, such as IL-6 and IL-10, are thought to be early measures of inflammatory response induced by surgical trauma. Local anesthetics via a nerve block have been demonstrated to attenuate the local inflammatory response [12]. For instance, previous animal experiments have demonstrated that C-fiber blockade may inhibit peripheral inflammation in the corresponding innervated zone [12, 13]. Furthermore, in postoperative patients, peripheral nerve blocks have been shown to attenuate postoperative inflammatory response in knee arthroplasty [14, 15]. The scalp is densely innervated with C-fibers (unmyelinated) and A-delta fibers (thinly myelinated) [16].

However, as far as we know, no study has attempted to investigate the impact of scalp nerve block on inflammatory response in the craniotomies.

Therefore, the goal of this prospective, randomized, controlled study was to compare the impact of scalp nerve block and local anesthetic infiltration with 0.75% ropivacaine on postoperative inflammatory response, intraoperative hemodynamic response, pain scores, and oxycodone consumption in the first 48 h postoperatively in patients undergoing craniotomy for cerebral aneurysms.

## Methods

### Patients

After written informed consent was obtained, 57 adult patients (ASA I or II, aged 18 to 65 years) who were scheduled for an elective craniotomy for surgical clipping of cerebral aneurysm located in the anterior cerebral circulation were included.

We did not enroll patients if they (1) had difficult surgical anatomy or multiple or giant aneurysms, (2) had a previous craniotomy incision, (3) had a history of allergy to opioids or local anesthetics, (4) had a history of drug dependence and alcohol abuse, or (5) could not understand a visual analog scale (VAS) or communicate [scheduled to be sedated postoperatively or a GCS (Glasgow coma score) < 14]. The first author (X.Y.) enrolled the patients in the study.

### Anesthesia, surgery and postoperative pain relief

The patients included in the current study were anesthetized by the same anesthesiologist and operated on by the same surgeon. Patients received 0.03 mg/kg IV midazolam as a preanesthetic medication, and 0.25 mg prophylaxis of postoperative nausea and vomiting (PONV) was achieved with palonosetron for all patients.

Anesthesia and monitoring were standardized for all patients. Electrocardiography, pulse oximetry, noninvasive blood pressure, end-tidal CO_2_ (ETCO_2_), nasopharyngeal temperature, and bispectral index (BIS) were continuously monitored during anesthesia and were recorded at fixed intervals of 5 min. Before anesthesia induction, four electrodes for measuring EEG signals were applied to the patient’s forehead, as recommended by the manufacturer (BIS Sensor, Covidien B.V. Zaltbommel, Netherlands), and were used to measure BIS values, signal quality index (SQI) and electromyography (EMG). BIS was displayed using an Aspect EEG monitor (A-2000, version 3.2; Aspect Medical Systems, Newton, M.A.). (We wiped clean the skin on the forehead with an alcohol-soaked skin wipe to lower the skin/electrode impedance. When SQI was greater than 50% the BIS values were considered valid. When SQI was less than 50% and the duration exceeded 20% of the total study time, all data for this patient was excluded.) General anesthesia was induced with 1.5–2.0 mg/kg propofol and 0.5–0.8 μg/kg sufentanil, and 0.2 mg/kg cis-atracurium was administered to facilitate orotracheal intubation. After intubation, we placed an arterial catheter to monitor mean arterial pressure (MAP) and collect a blood sample, and an intravenous catheter was placed in the right jugular vein. After intubation, all patients were ventilated mechanically with a tidal volume of 8 ml/kg, and the respiratory rate was adjusted accordingly to maintain 35–40 mmHg of paCO2 (partial pressure of carbon dioxide in the artery). Then, 4–9 mg/kg/h propofol was infused continuously to maintain anesthesia. The infusion rate of propofol was adjusted to keep the BIS within 40–60. Remifentanil was adjusted according to the degree of surgical manipulation (0.1–0.5 μg/kg/min). If intraoperative MAP and heart rate (HR) increased by more than 20% from baseline, supplemental doses of 0.5 μg/kg remifentanil were administered, and the infusion rate of remifentanil was increased by 0.05 μg/kg/min. If the increased MAP and HR did not respond to higher remifentanil infusion rate, nicardipine or esmolol was administered, as appropriate. Considering intraoperative neurophysiological monitoring, we did not administer additional neuromuscular blocker during the surgery. Normothermia was maintained throughout the surgery. Volume was replaced by 0.9% sodium chloride and 130/0.4 hydroxyethyl starch. Patients were extubated after the operation in the post-anesthetic care unit (PACU) when awake and in a neurologically stable condition before being transferred to the neurosurgical intensive care unit (NICU).

All patients were administered oxycodone (0.1 mg/kg) 30 mins before the end of surgery. Oxycodone was also used as rescue analgesia in the first 48 h postoperatively. Pain was evaluated with visual analogue scale (VAS) scores from 0 to 10 (0 = no pain, 10 = worst pain). If a patient reported a VAS more than 3, an intravenous injection of 2 mg of oxycodone was given by a nurse as the rescue analgesia. This dose was administered at 15-min intervals until VAS was less than 3. Oxycodone consumption in the first 48 postoperative hrs and the time to first rescue requirement were recorded.

### Randomization

A randomization list was generated, and patients were assigned consecutively to one of three groups by the third author (R.D.), who was not involved in patient care. Scalp nerve block was performed in Group S, local anesthetic infiltration was performed in Group I, and patients in Group C only received sufentanil, remifentanil and oxycodone as analgesics during the intraoperative period.

The patients and the second author (J.M.) who followed the hemodynamic response to skin incision, drew the blood samples, and recorded postoperative pain scores and rescue analgesic consumption were blinded in every case.

### Scalp nerve block and local anesthetic infiltration

In Group S, the scalp blocks were performed bilaterally with 15 mL of 0.75% ropivacaine 10 mins before the incision by the anesthesiologist using the method described by Pinosky et al. [17]. The supraorbital and supratrochlear nerves emerge from the orbit, and a 25-gauge needle was introduced above the eyebrow perpendicular to the skin. These nerves were blocked bilaterally with 4 mL of 0.75% ropivacaine. The zygomatico-temporal nerve emerges lateral to the orbit, equal to the position of pterion, and this nerve was blocked bilaterally with 2 mL of 0.75% ropivacaine. The auriculotemporal nerve was blocked bilaterally 1.5 cm anterior to the ear at the level of the tragus, the needle was introduced perpendicularly to the skin and infiltration was performed deep into the fascia and superficially as the needle was withdrawn. Care must be taken to avoid destroying the superficial temporal artery. These nerves were blocked bilaterally with 2 mL of 0.75% ropivacaine. The greater, lesser, and third occipital nerves were blocked bilaterally with 7 mL 0.75% ropivacaine injected using a 22-gauge needle along the superior nuchal line, approximately halfway between the occipital protuberance and the mastoid process.

In Group I, the surgical incision sites were infiltrated with 15 mL of 0.75% ropivacaine 10 mins before the incision by the neurosurgeon. Neither scalp blocks nor local infiltration was performed in Group C.

### Outcome measurements

Patient characteristics, type of aneurysm, duration of anesthesia and surgery, total dose of propofol and remifentanil, fluid balance, and number of patients who used nicardipine or esmolol were documented.

Plasma levels of CRP, IL-6, and IL-10 were measured in pre-, intra- and. postoperative periods, and EDTA arterial blood samples were collected to measure the concentrations of CRP, IL-6, and IL-10 in plasma at the following time points: (Baseline) before the induction of anesthesia, (6H) 6 h after incision, and (12H) 12, (24H) 24, (48H) 48, and (72H) 72 h postoperatively. After centrifugation, plasma samples were stored at − 80 °C until analysis. The serum levels of CRP, IL-6, and IL-10 were measured using enzyme-linked immunosorbent assay (Elabscience Biotechnology Co. Ltd., Wuhan, China) following the manufacturer’s instructions. The detection levels of cytokines and inflammatory mediators in the assays were 0.4 ng/mL for CRP, 4 pg/mL for IL-6, and 1 pg/mL for IL-10.

MAP and HR were recorded just before anesthesia induction (T1); 5 mins after induction (T2); 5 mins after skin incision (T3); 2 mins (T4) and 5 mins (T5) after the incision; during dura opening (T6); and at the end of surgery (T7).

Postoperative VAS, cumulative oxycodone consumption and postoperative pain control-related side effects such as postoperative nausea and vomiting (PONV), infection and pruritus were recorded 2, 4, 8, 12, 24, and 48 h after recovery of consciousness. Additionally, the time intervals from patient recovery to the first use of oxycodone and consumption of oxycodone 48 h postoperatively were recorded.

The primary endpoint of the current study was the effect of scalp nerve block and local infiltration with 0.75% ropivacaine on postoperative inflammatory response in patients undergoing craniotomy for cerebral aneurysms. The secondary endpoints were the effects of scalp nerve block and local infiltration on the hemodynamic response to skin incision, postoperative pain intensity, cumulative oxycodone consumption and pain control-related side effects 48 h postoperatively.

### Statistical analysis

The data are expressed as the mean ± standard deviation (SD), median and interquartile range (IQR, 25–75% percentile) or number (%). The Kolmogorov–Smirnov test was used to assess the normality and homogeneity of all the variables.

Continuous variables were presented as the mean ± SD and analyzed using one-way ANOVA with post hoc correction for multiple comparisons (Bonferroni correction) to determine differences among groups. Categorical variables were described as numbers (%) and were compared using chi-square tests. Biological data (CRP, IL-6, and IL-10 levels) and hemodynamic data (HR, MAP) were compared among groups and over time using repeated-measures ANOVA. Nonnormally distributed continuous variables, such as pain scores, were presented as median and interquartile range (IQR, 25–75 percentile) and were analyzed with nonparametric tests (Kruskal–Wallis test and Mann-Whitney U-test with Bonferroni correction).

On the basis of a previous study [7], we assumed that a difference of 20% in MAP was clinically relevant, and setting α equal to 0.05 and β equal to 0.2, we calculated a necessary sample size of 15 patients per group. Values of *P* < 0.05 were considered significant. SPSS statistical software, version 21.0 (SPSS, Inc., Chicago, Illinois, USA), was used for data analysis.

## Results

### Patient demographics and perioperative characteristics

**Fig. 1:**
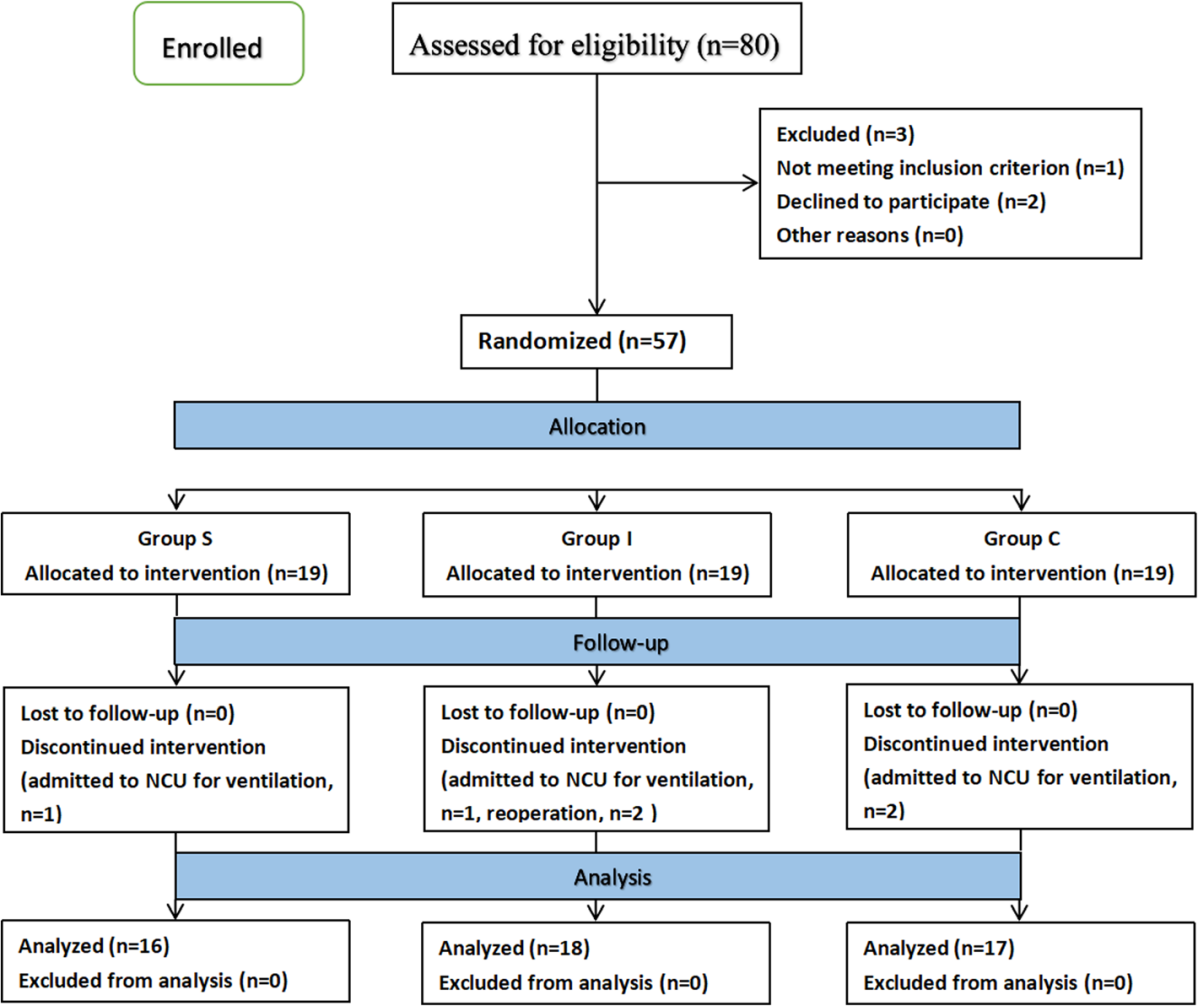
CONSORT flow diagram

Fifty-seven patients agreed and were randomized into the study, and 6 patients were excluded from the study after randomization due to unexpected sedation after surgery and delayed extubation (*n* = 5, 1 in Group C, 2 in Group I and 2 in Group S) or reoperation (*n* = 1 in group I). Thus, the remaining 51 patients were analyzed (18 in Group S, 16 in Group I and 17 in Group C). The consort diagram showed the flow of participants through each stage of a randomized trial (Fig. 1).

The three groups were similar in age, gender, BMI, ASA, type of aneurysm, duration of operation, duration of anesthesia, cumulative dose of propofol, total loss of blood, urine volume and infusion volume. There were significant differences among the study groups (*F* = 205.377; *P* < 0.001, Table 1) for the cumulative dose of remifentanil. Patients in Group C received a higher cumulative dose of remifentanil (4.59 ± 0.64 mg) compared to Group I (3.67 ± 0.38 mg) and Group S (1.40 ± 0.38 mg) (*P* < 0.001, *P* < 0.001, respectively). Patients in Group I consumed more remifentanil during the operation than patients in Group S (*P* < 0.001).

**Table 1:** Patient Demographics and Perioperative Characteristics

Characteristic	Group C (*n* = 17)	Group I (*n* = 16)	Group S (*n* = 18)	*P* values
Age (years)	54.41 ± 6.62	55.19 ± 5.94	55.94 ± 5.14	NS
Gender (M/F)	4/13	4/12	6/12	NS
BMI (kg/m^2^)	22.48 ± 1.30	22.54 ± 0.80	22.51 ± 0.90	NS
ASA (I/II)	7/10	8/8	8/10	NS
Type of aneurysm (number%)				NS
Middle cerebral artery	11 (64.6%)	12 (75%)	13 (72.2%)	
Anterior communicating artery	2 (11.8%)	0	1 (5.6%)	
Posterior communicating artery	2 (11.8%)	2 (12.5%)	3 (16.6%)	
Anterior cerebral artery	2 (11.8%)	2 (12.5%)	1 (5.6%)	
Duration of operation (min)	169.41 ± 44.75	161.88 ± 36.69	155.56 ± 40.94	NS
Duration of anesthesia (min)	252.94 ± 42.65	248.44 ± 36.96	240.28 ± 41.53	NS
Total dose of propofol (mg)	1854.65 ± 375.03	1820.88 ± 233.17	1716.33 ± 206.67	NS
Total dose of remifentanil (mg)	4.59 ± 0.64	3.67 ± 0.38	1.40 ± 0.38	0.000^*^
Total loss of blood (ml)	370.59 ± 161.11	393.75 ± 166.21	305.56 ± 93.76	NS
Urine volume (ml)	1135.29 ± 337.16	1218.75 ± 335.10	1055.56 ± 261.72	NS
Infusion volume (ml)				
Crystalloids (ml)	1361.76 ± 644.58	1468.75 ± 618.30	1147.22 ± 269.24	NS
Colloids (ml)	1161.76 ± 264.30	1109.38 ± 240.98	1111.11 ± 213.90	NS
Number of use of nicardipine(%)	8 (47.1%)	3 (18.8%)	1 (5.6%)	0.017^#^
Number of use of esmolol(%)	0	0	0	NA

Values are expressed as mean ± SD or number of patients(%)

The differences among groups were not significant except for the consumption of remifentanil and the use of nicardipine

*Group C* Control group, *Group I* Local anesthesic infiltrationgroup, *Group S* Scalp nerve block group

*Abbreviations*: *ASA* American society of anaesthesiologists, *NA* Not applicable, *NS* Not significant

^#^*P* < 0.05 for Group I and Group S compared with group C. *P < 0.05 among three groups

Additionally, 8 patients (47.1%) in Group C, 3 patients (18.8%) in Group I, and 1 patient (5.6%) in Group S used nicardipine during the operation. Nicardipine administration was different among the three groups (*P* = 0.017 according to Fisher’s exact test, Table 1), and nicardipine was less frequently required in Group S than in group C (*P* = 0.007, Table 1).

### Plasma concentrations of CRP, IL-6, and IL-10

**Fig. 2:**
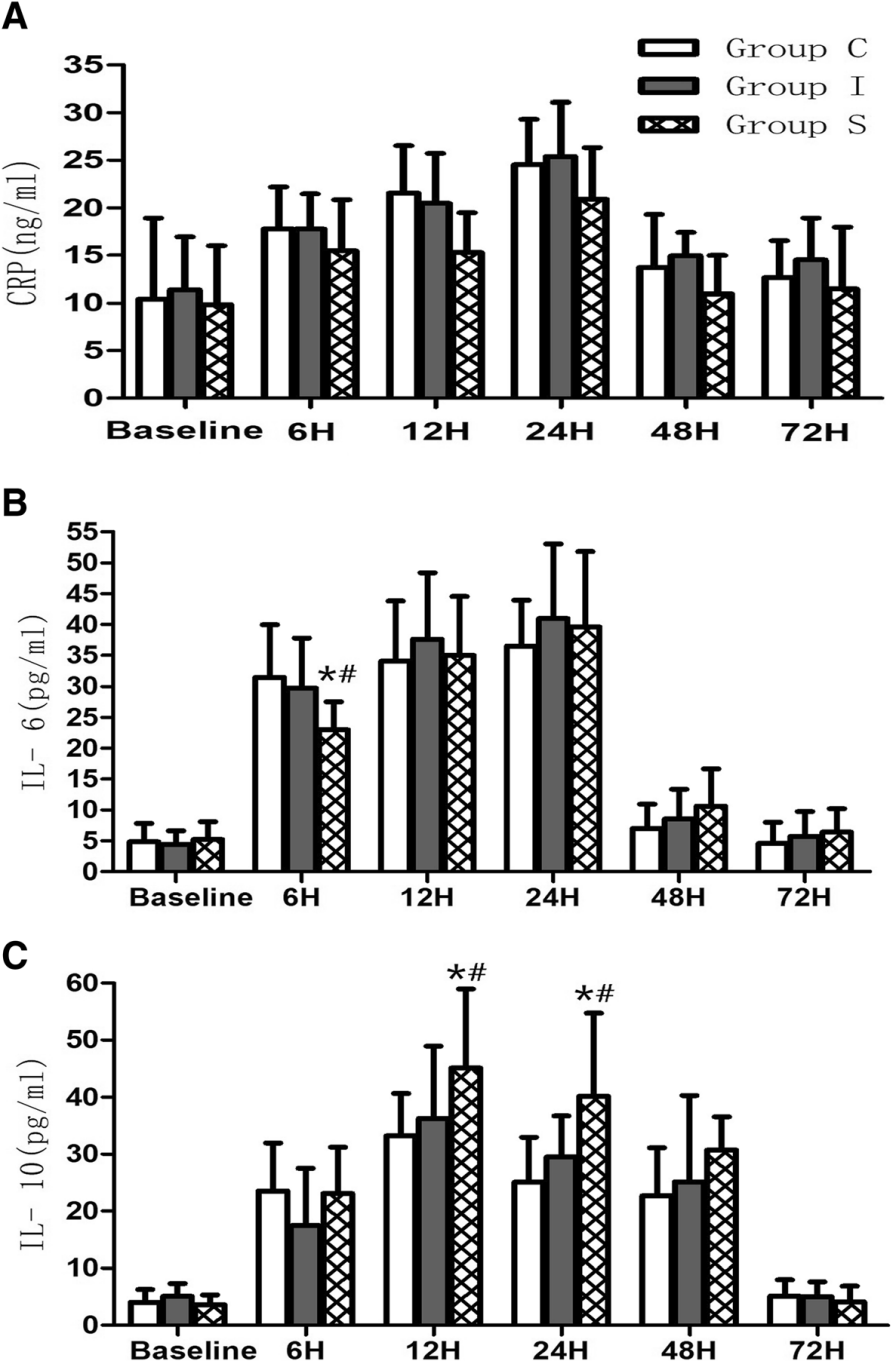
Concentrations of **a** C-reactive protein (CRP), **b** interleukin-6 (IL-6), and **c** interleukin-10 (IL-10) preoperatively (Pre-op) and 6, 12, 24, 48 and 72 h postoperatively in the three groups studied. Group C: control group, Group I: local anesthetic infiltration group, Group S: scalp nerve block group. ^*^P < 0.05, compared to Group C, ^#^*P* < 0.001, compared to Group I

Plasma concentrations of CRP, IL-6, and IL-10 at all time points are displayed in Fig. 2. Plasma CRP levels significantly changed with time in the three groups (main effect of time: F(3.874, 185.944) = 108.039, *P* < 0.001). In all groups, CRP levels increased 24 h after surgery, reaching maximum values at 24 h, and subsequently decreased gradually until 72 h after the operation (Fig. 2a). Additionally, there was no significant interaction between analgesia mode and time in plasma levels of CRP (group-time interaction: F(7.748, 185.944) = 1.43, *P* = 0.069). Although plasma CRP was not significantly different among the three groups, there was a tendency for lower CRP in Group S compared to Groups C and I 12 and 24 h postoperatively (Fig. 2a).

The same trend applied to IL-6 levels. There was a significant difference over time among the three groups in plasma IL-6 values (main effect of time: F(2.238, 107.447) = 303.761, *P* < 0.001). IL-6 values increased 24 h after surgery, reaching peak at 24 h and decreasing gradually until 72 h after the operation (Fig. 2b). Moreover, there was a significant interaction between analgesia mode and time on plasma levels of IL-6 (group-time interaction: F(4.477, 107.447) =2.47, *P* = 0.043), and patients in Group S had lower IL-6 6 h postoperatively compared with those in Groups C and I (*P* = 0.001, and *P* = 0.009, respectively) (Fig. 2b).

Plasma IL-10 levels significantly changed with time in all three groups (main effect of time: F(3.189, 153.067) = 198.014, *P* < 0.001). In all three groups, IL-10 levels increased 48 h after surgery, reaching maximum values at 12 h, and subsequently decreased gradually until 72 h after the operation (Fig. 2c). There was a significant interaction between analgesia mode and time on plasma levels of IL-10 (group-time interaction: F(6.378, 153.067) = 5.107, *P* < 0.01). Furthermore, patients in Group S had higher IL-10 levels 12 and 24 h postoperatively compared with those in Groups C and I (12 h: *P* = 0.012, and *P* < 0.001, 24 h: *P* = 0.011, and *P* < 0.001, respectively) (Fig. 2c).

### Hemodynamic parameters (HRs and MAPs)

**Fig. 3:**
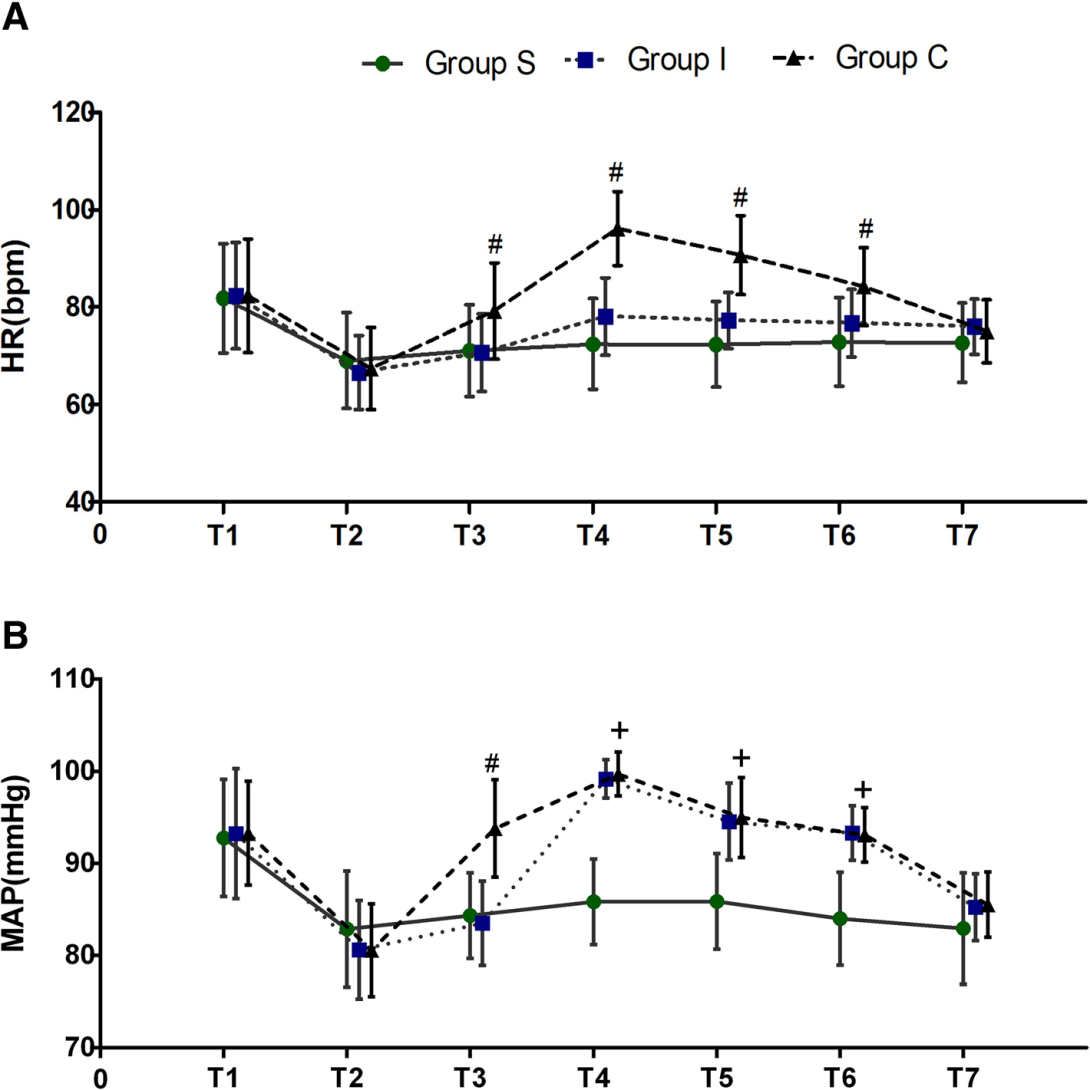
Comparison of HR and MAP changes during surgery. T1: before anesthesia induction, T2: 5 mins after induction, T3: 5 mins after skin incision, T4: 2 mins after the incision, T5: 5 mins after the incision, T6: during dura opening, and T7: the end of the surgery. Group C: control group, Group I: local anesthetic infiltration group, Group S: scalp nerve block group. ^#^*P* < 0.05, for Group C compared with Groups I and S, ^+^*P* < 0.001 for Groups I and C compared with Group S

HRs were significantly lower in Groups I and S compared to Group C at T3, T4, T5 and T6 (group-time interaction: F(3.46, 166.075) =86.081, *P* < 0.001). Post hoc analysis showed significant differences during skin incision (T3) (*P* = 0.03, *P* = 0.035, respectively) and the second (T4) (*P* < 0.001, *P* < 0.001, respectively) and fifth minutes (T5) after incision (*P* < 0.001, *P* < 0.001, respectively) and during dura opening (T6) (*P* = 0.032, *P* < 0.001, respectively). There were no significant differences in HRs between Group S and Group I at any time point (*P* > 0.05) (Fig. 3a).

There were significant differences in MAP among the three groups at T3, T4, T5 and T6 (group-time interaction: F(6.995, 167.883) = 24.192, *P* < 0.001). Post hoc analysis showed that MAPs were significantly lower in Group I and Group S compared to Group C during skin incision (T3) (Group S vs. Group C: *P* < 0.001, Group I vs. Group C: *P* < 0.001). Additionally, MAPs in Group S were significantly lower than those in Group I and Group C at the second (T4) and fifth minutes (T5) after the incision and during dura opening (T6) (T4, T5 and T6: Group S vs. Group I, *P* < 0.001; Group S vs. Group C, *P* < 0.001). However, there were no significant differences in MAPs between Groups I and C at T4, T5 and T6 (*P* > 0.05) (Fig. 3b).

### Postoperative pain scores and oxycodone consumption

**Fig. 4:**
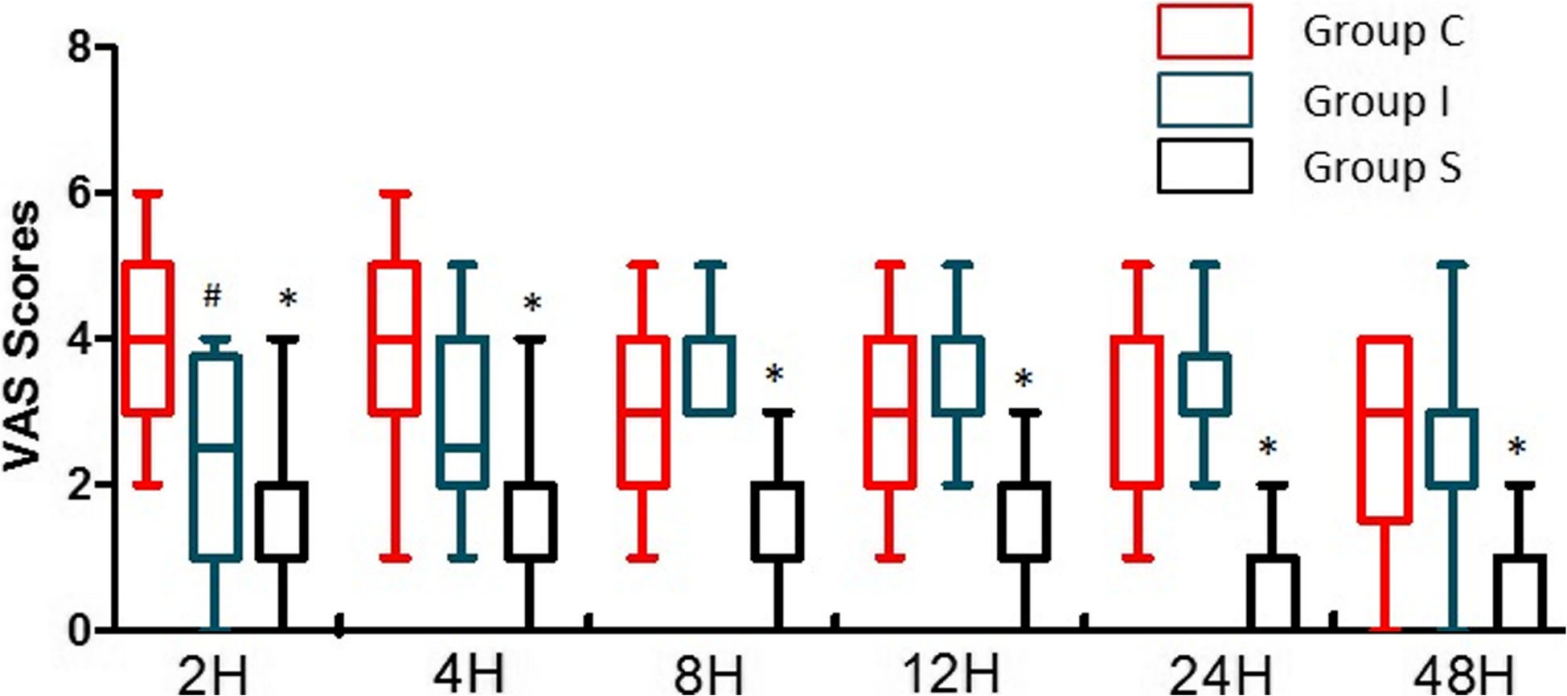
Comparison of VAS scores postoperatively for all three groups. Group C: control group, Group I: local anesthetic infiltration group, Group S: scalp nerve block group. ^#^*P* < 0.05, Compared to Group C, ^*^P < 0.001 for Group S compared with Groups I and C

The VAS scores were significantly lower in Group S than in Group C and Group I 2, 4, 8, 12, 24 and 48 h postoperatively (*P* < 0.001, respectively). However, Group I only had lower VAS scores compared to Group C 2 h after surgery (*P* = 0.026) (Fig. 4).

**Fig. 5:**
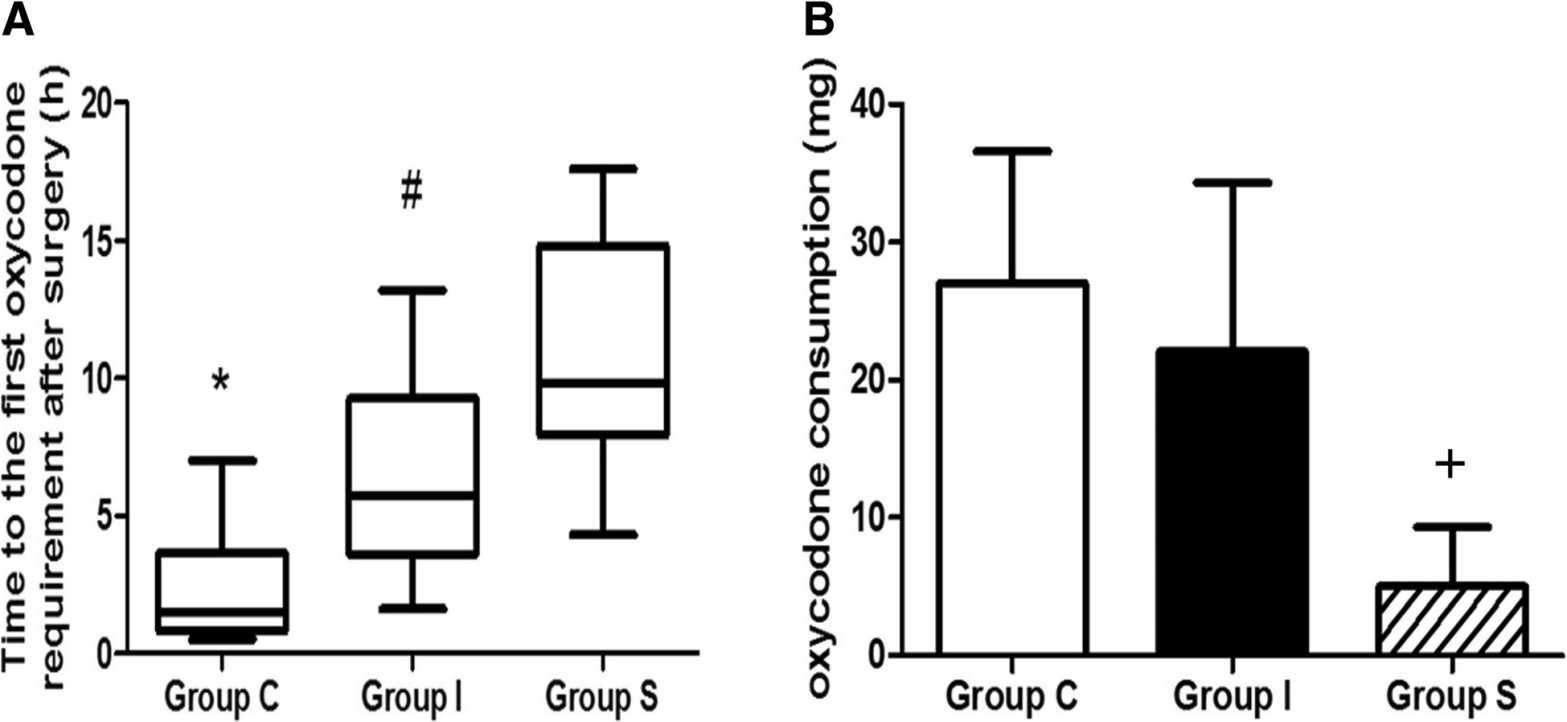
Comparison of **a** first patient request for rescue analgesia and **b** oxycodone consumption during the first 48 postoperative hrs. Group C: control group, Group I: local anesthetic infiltration group, Group S: scalp nerve block group. ^*^P < 0.01 for Group C compared with Groups I and S, ^#^*P* < 0.05 for Group I compared with Group S, ^+^*P* < 0.001 for Group S compared with Groups C and I

The time intervals from patient recovery to the first use of oxycodone in Groups I and S were significantly longer than those in Group C [5.75(3.58–9.28) and 9.85 (7.93–14.83) vs. 1.5(0.8–3.65) hrs, *P* = 0.009, and *P* < 0.001, respectively]. In addition, the first use of oxycodone in Group S was significantly longer than that in Group I (*P* = 0.018) (Fig. 5a).

Oxycodone consumption after 48 h was significantly higher in Groups C and I than in Group S (27 ± 9.6 and 22.06 ± 12.24 vs. 5.01 ± 4.3 mg, *P* < 0.001, *P* < 0.001, respectively). There was no significant difference in oxycodone consumption between Groups I and C (*P* = 0.386) (Fig. 5b).

### Pain control-related adverse events during the study period

Adverse events 48 h after surgery, such as respiration depression, cutaneous pruritus, subcutaneous hematomas, scalp infection, and local anesthetic toxicity were not observed. However, the incidence of PONV was significantly different among the three groups (*P* = 0.017, Table 2.). Five patients (29.4%) in Group C, 4 patients (25%) in Group I and 2 patients (11.1%) in Group S suffered from PONV, and PONV occurred less frequently in Group S than Group C (*P* = 0.012, Table 2). There were no significantly differences in the incidence of fever or dizziness among groups (*P* = 0.721, *P* = 0.462, respectively, Table 2).

**Table 2:** Pain control related adverse events during the study period (48 h after surgery)

	Group C (*n* = 17)	Group I (*n* = 16)	Group S (*n* = 18)	*P* values
Fever	1 (5.9%)	2 (12.5%)	1 (5.6%)	NS
Nausea and Vomit*	5 (29.4%)	4 (25%)	2 (11.1%)	0.017*
Dizziness	1 (5.9%)	0	1 (5.6%)	NS
Respiration depression	0	0	0	NA
Cutaneous pruritus	0	0	0	NA
Subcutaneous haematomas	0	0	0	NA
Scalp infection	0	0	0	NA
Local anesthetic toxicity	0	0	0	NA

Values are given as number of patients(%)

*Group C* Control group, *Group I* Local anesthesic infiltration group, *Group S* Scalp nerve block group

*Abbreviations*: *NA* Not applicable, *NS* Not significant

^*^P < 0.05 comparison among three groups

## Discussion

This prospective, randomized, controlled study demonstrated that scalp nerve block with 0.75% ropivacaine had a modest preventive effect on postoperative inflammation demonstrated by lower IL-6 concentrations in plasma 6 h after craniotomy for cerebral aneurysms and reduced CRP levels and increased IL-10 levels 12 and 24 h postoperatively. Scalp nerve block blunted the hemodynamic response to skin incision better than local anesthetic infiltration or routine anesthesia. Additionally, both the scalp nerve block and local anesthetic infiltration decreased remifentanil consumption during the operation compared to the control group, and the scalp nerve block group had lower postoperative pain intensity, longer duration before the first dose of oxycodone, less consumption of oxycodone and lower incidence of PONV 48 h postoperatively than the local anesthetic infiltration group and the control group.

Substances released by sensory nerve endings produce inflammation in the target tissue, which is a progress of neurogenic inflammation response [18]. The key point of neurogenic inflammation is the activation of the primary afferent, which cause dorsal root reflexes in the spinal cord [19]. Blocking the nerve through local anesthetics can reduce the release of substances, such as substance P and calcitonin gene–related peptide, and block neural transmission at the site of tissue injury, thereby alleviating the neurogenic inflammatory response [20]. However, the exact mechanisms are still largely unclear.

Therefore, it is possible that in the current study, the reduced concentrations of pro-inflammatory cytokines CRP and IL-6, as well as the increased concentration of anti-inflammatory cytokine IL-10 in plasma, compared to local anesthetic infiltration and routine analgesia, were related to the local anti-inflammatory effects of scalp nerve block. It is possible that scalp nerve block has a greater deafferentation effect than local anesthetic infiltration that prevents the development of peripheral and systemic inflammation. There is also evidence suggesting that inflammation and pain are related [21]. In the current study, patients in the scalp nerve block group had lower postoperative pain intensity and longer duration before the first dose of oxycodone than patients in the local anesthetic infiltration and routine analgesia groups, supporting our findings that scalp nerve block inhibited craniotomy-induced inflammation.

The effects of neuraxial blockade with local anesthetics on postoperative inflammatory response are still controversial. For example, a previous study suggested that a combined continuous lumbar plexus and sciatic nerve blocks with 0.2% ropivacaine contributed to the attenuation of postoperative inflammatory response, which was demonstrated by decreased CRP and IL-6 concentrations in plasma 24 and 48 h postoperatively [14]. Another clinical study using more extensive nerve blocks, such as epidural analgesia, have also indicated attenuated ex vivo pro-inflammatory cytokine IL-6 and anti-inflammatory cytokine IL-10 production after visceral surgery [22]. However, Moore and coworkers found that the circulating CRP and IL-6 response to pelvic surgery was unaffected by extradural analgesia [23]. This finding is in contrast with the present study where the scalp nerve block inhibited CRP and IL-6. Such discrepancies could originate from the nerve block technique, the type of surgery, and probably the assay used to measure the inflammatory mediator concentration (i.e., ex vivo or in vivo assays).

Of note is that, in our study, scalp nerve block only had modest anti- inflammatory effects. Previous studies have indicated the impacts of remifentanil on systemic inflammation. For example, remifentanil has been indicated to reduce plasma IL-6 levels on the seventh day after abdominal surgery [24]. It has also been demonstrated to inhibit exaggerated inflammation after cardiac surgery with cardiopulmonary bypass [25]. In the present study, we found that patients in the scalp nerve block group consumed less remifentanil during the operation than patients in the local anesthetic infiltration group and the routine analgesia group. Therefore, remifentanil requirements may be a confounding factor that hampered the interpretation of the effects of different analgesic modalities on the inflammatory response caused by craniotomy. Furthermore, our study might have been underpowered because of the small study group size, which could also explain the modest anti-inflammatory effects of scalp nerve block. Collectively, our findings suggest a potential anti-inflammatory effect of scalp nerve block with 0.75% ropivacaine, pending further investigations.

Acute increases in MAP and HR could be deleterious for neurosurgical patients with cerebral aneurysm, given that acute arterial hypertension and tachycardia may result in ruptured cerebral aneurysms. In the current study, we found that scalp nerve block blunted hemodynamic response to skin incision and dura opening better than local anesthetic infiltration or routine anesthesia. These results are in line with the study by Geze1 et al. [7], in which scalp nerve block with 0.5% bupivacaine was shown to be better at blunting the hemodynamic response to strong nociceptive stimulus, such as head pinning, than local infiltration or routine analgesia [7]. However, the it has also been reported that local infiltration promotes intraoperative hemodynamic stability in patients undergoing craniotomy [26–28]. Our study is inconsistent with these studies. The discrepancy may be explained by different local anesthetic used (bupivacaine vs. ropivacaine), the time point studies or the nociceptive stimulus.

Both scalp nerve block and local infiltration have been demonstrated to reduce VAS scores and opioid requirements after surgery. For example, a meta-analysis of scalp blocks demonstrated not only a significant reduction in VAS scores 2, 4, 6 and 8 h after the operation, with the most significant mean reduction occurring 1 h after surgery, but also a decrease in opioid requirements over the first 24 h postoperatively. However, few studies have compared the effects of scalp nerve block and local infiltration on postoperative pain control. In the current study, we found that the scalp nerve block group had lower postoperative pain intensity, longer duration of time before the first dose of oxycodone, less consumption of oxycodone and lower incidence of PONV 48 h postoperatively than the local infiltration group and control group. Our study is consistent with the study by Hwang et al. [29], showing that scalp blocks with 0.75% levobupivacaine effectively lowered postoperative pain and PCA consumption 72 h after patients underwent frontoparietal craniotomy for aneurysm clipping compared to routine analgesia. In our study, the beneficial effect of the scalp block lasted longer than the expected duration of action. This phenomenon could be explained by preemptive analgesia [30], which commences before surgery and continues in the postoperative period, preventing the establishment of peripheral and central sensitization since we performed the scalp block prior to scalp incision [31].

It is noteworthy that in our study, PONV occurred less frequently in the scalp nerve block group than the local infiltration group and the control group. It is possible that the lower incidence of PONV in the scalp nerve block group was related to less intraoperative remifentanil consumption and postoperative oxycodone use.

The current study has several limitations. First, 0.75% ropivacaine (15 mL, 112.5 mg) was used for the scalp nerve block and local infiltration, but the plasma concentration was not measured, and the maximum recommended dose of ropivacaine is 225 mg with or without epinephrine [32]. Furthermore, in our study, no patient developed side effects related to local anesthetic toxicity. Second, we did not apply isotonic sodium chloride for the scalp nerve block or local infiltration in the control group. Thus, we could not rule out the effects of injection stress. Third, in the present study, we only focused on the effects of different analgesic modalities on systemic inflammatory response but not local inflammatory response at the site of tissue injury. Fourth, because the impact of different analgesic modalities on inflammatory response to craniotomy has been rarely reported, in the current pilot study, the sample size calculation was done based on MAP and not inflammatory markers in response to craniotomy. Accordingly, the present study may not be sufficiently powered to provide strong evidence for the effects of scalp nerve block and local anesthetic infiltration on inflammatory response, hemodynamic response, or postoperative pain control in patients undergoing craniotomy due to the small sample size. A larger study with adequate power is needed to validate our results. Finally, in the current study, we only evaluated the effect of the ropivacaine scalp block on acute pain after craniotomy (48 h postoperatively) but not chronic postcraniotomy headache. These limitations indicate the need for further investigations.

## Conclusions

In conclusion, the present study shows that scalp nerve block with 0.75% ropivacaine attenuated inflammatory response to craniotomy for cerebral aneurysms, blunted hemodynamic response to scalp incision, and controlled postoperative pain better than local anesthetic infiltration or routine anesthesia. Scalp nerve block should be considered in conjunction with general anesthesia for aneurysm clipping. Scalp nerve block might exert potential anti-inflammatory neuroanesthetic effects pending further investigations.

## Data Availability

The datasets generated and analyzed in the current study are available from the corresponding author on reasonable request.

## Abbreviations

BIS: Bispectral index
CRP: C-reactive protein
EMG: Electromyography
ERAS: Enhanced recovery after surgery
ETCO2: End-tidal CO2
GCS: Glasgow coma score
HR: Heart rate
ICP: Increased intracranial pressure
IL-6: Interleukin-6
IQR: Interquartile range
LA: Local anesthetic infiltration
MAP: Mean arterial pressure
NICU: Neurosurgical intensive care unit
paCO2: Partial pressure of carbon dioxide in the artery
PACU: Post-anesthetic care unit
PONV: Postoperative nausea and vomiting
SNB: Scalp nerve block
SQI: Signal quality index
VAS: Visual analog scale
